# Photodegradation of octylisothiazolinone and semi-field emissions from facade coatings

**DOI:** 10.1038/srep41501

**Published:** 2017-01-27

**Authors:** Ulla E. Bollmann, Greta Minelgaite, Michael Schlüsener, Thomas A. Ternes, Jes Vollertsen, Kai Bester

**Affiliations:** 1 Aarhus University, Department of Environmental Science, Frederiksborgvej 399, 4000 Roskilde, Denmark; 2 Aalborg University, Department of Civil Engineering, Sofiendalsvej 11, 9200 Aalborg SV, Denmark; 3 German Federal Institute for Hydrology, Am Mainzer Tor 1, 56068 Koblenz, Germany

## Abstract

Amongst others, 2-octyl-isothiazol-3(2 *H*)-one (OIT) is used as film preservative in water-based polymer resin paints and renders to prevent the growth of moulds and bacteria. It is known that biocides leach from facades with rainwater and end up in the environment via stormwater runoff. In the present study the leaching and fate of OIT used in facade coatings was determined under natural conditions. Potential phototransformation products were initially identified in laboratory experiments using UV-light. Afterwards, the leaching of OIT and seven degradation products were studied on artificial walls equipped with organic top coatings formulated with OIT. A mass balance, including the leached and remaining amounts of OIT and its seven transformation products, can explain up to 40% of the initial amount of OIT. The OIT remaining in the material after 1.5 yr is by far the largest fraction. The study shows that in the assessment of biocides in coating material, transformation products need to be taken into account both in leachate and remaining in the material. Furthermore, in case of volatile degradation products, the emissions to air might be relevant.

Isothiazolin-3-ones are a widely used group of compounds to prevent the growth of moulds and fungi and, hence, belonging to the group of biocidal compounds regulated as such by the EU^1^,^2^. They are used in several household and industrial products, such as liquid cooling and processing systems, metal working fluids, fibre, leather and rubber material and last but not least in cosmetics and toiletries. Moreover, they are used as in-can as well as film preservatives in water-based polymer resin paints and renders. While 2-methylisothiazol-3(2 *H*)-one (MI), 5-chloro-2-methylisothiazol-3(2 *H*)-one (CMI) or benzo[*d*]isothiazol-3(2 *H*)-one (BIT) are used as in-can preservatives to increase shelf life, 2-octylisothiazol-3(2 *H*)-one (OIT) and 4,5-dichloro-2-octylisothiazol-3(2 *H*)-one (DCOIT) are typically added as film preservatives to protect the final coating film of indoor and outdoor walls. Additionally, DCOIT is a commonly used antifouling biocide to protect boat and ship hulls.

Several monitoring studies showed that biocides leach in substantial amounts to the environment and can be detected in stormwater^3^,^4^,^5^, wastewater^6^,^7^,^8^,^9^ and surface waters^10^,^11^,^12^,^13^,^14^. Especially the application as preservative in water-based polymer resin paints and renders, which are commonly applied on *external thermal insulation composite systems (ETICS*), are a direct source for biocides in the environment. It is known, that OIT and other biocides leach from the facade material when it gets in contact with rainwater^15^. Laboratory experiments have shown that the water contact time seems to have an important influence on the leaching. Hence, a diffusion controlled process is expected^3^,^16^,^17^,^18^. However, the amount of total leached OIT and other biocides can be increased by insertion of drying cycles^17^ and is even higher if the material stays wet between leaching events^18^. In laboratory^19^ as well as in long-term exposure studies^15^,^20^,^21^ it was shown that biocides as terbutryn or diuron leach predominantly within the first half year to a year of exposure and decrease considerably later on, although only a small fraction of the initial content is leached over the first five years of exposure (<13%)^20^. While polymer resin renders are preserved by the addition of biocides prior to application, concrete surfaces are often treated with insecticides or herbicides during their lifetime^22^. Although applied as spray, and hence, initially loosely attached to the surface, similar leaching profiles have been observed for the wash-off of herbicides and insecticides from concrete surfaces^22^,^23^, as described by Wangler *et al*. for polymer resin render^17^.

While the analysis of the fraction remaining in the render after the exposure period revealed significant amounts of OIT remaining in the render materials, it showed gaps of more than 70% in the mass balance^15^,^20^. Hence, other release or removal processes need to be taken into consideration as well. Photodegradation is expected to be the most common dissipation pathway^15^,^20^,^21^,^22^, as biocides need to migrate from the solid material to the surface water film to be active and, hence, are susceptible to sunlight when reaching the coating surface. For future material design, knowledge on the photodegradation is needed to decrease losses of the active compounds. It has to be mentioned that likewise fast degradation in the environment is desired by e.g. the European biocidal product regulation^1^.

To the authors’ knowledge, no study has been published on the photodegradation of OIT so far. However, some studies have been published on photodegradation of MI and CMI as well as DCOIT. As main transformation pathway, the cleavage of the isothiazolinone ring structure and successively formation of *N*-methyl/octyl malonamic acid is described. Further oxidation leads to *N*-methyl/octyl oxamic acid, *N*-octylacetamide, *N*-octyl carbamic acid, 2-chloro-*N*-ocylcarbamoyl-1-ethene-sulfonic acid, acetic acid, formic acid and mineralization^24^,^25^,^26^. The photodegradation products for MI, CMI and DCOIT are similar to the ones caused by biological degradation^25^,^26^,^27^. Sakkas *et al*.^24^ tentatively identified a minor fraction of 4,5-dichloro-3-octylthiazol-2(3 *H*)-one, which they refer to a photo isomerization pathway according to Rokach and Hamel^28^. Photodegradation half-life of DCOIT by natural sunlight in fresh waters is determined to be 130–155 h^24^. Kandavelu *et al*.^29^ tested the photocatalytic degradation of MI and CMI in water in the presence of nanocrystalline titanium dioxide (the photocatalytic active one as well as the inactive paint grade one) and determined that paint grade titanium dioxide is not having any influence on the photodegradation of isothiazol-3(2 *H*)-ones.

No data is available, linking the presence of OIT in facade coatings to photodegradation on facade surfaces and thus linking leaching and phototransformation of OIT under realistic conditions. In the present study the environmental fate of 2-octylisothiazol-3(2 *H*)-one (OIT) added as a film preservative to polymer resin facade coatings was tested. Potential transformation products were identified in photodegradation experiments with OIT dissolved in water using UV-light. Afterwards, the mass balance of OIT was determined on a set of six artificial walls equipped with organic top coatings (Fig. 1) formulated with OIT exposed to natural weather and thus natural sunlight. Over 19 months the run-off water was collected event-resolved. Both the run-off as well as the remaining fractions of OIT and its transformation products were analysed in the render at the end of the experiment. Such mass balances of OIT have never been published before.

## Results

### Identification of possible OIT photodegradation products in laboratory experiments

**Figure 2:**
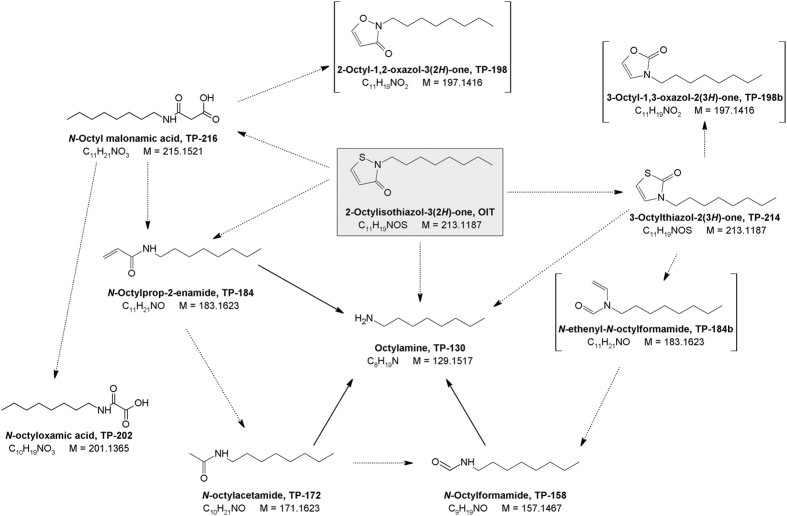
Proposed photodegradation pathway of OIT in water [in brackets: suggested compounds based only on HR-MS data, not validated by analytical standard].

The UV-irrigation of OIT led to degradation products with the following signals in electrospray positive ionization (ESI+) scans: 214.1252 Da, 158.1527 Da, 198.1486 Da, 184.1701 Da, 172.1696 Da, 130.1954 Da and 216.1595 Da. In the following, the observed peaks are named according to their nominal mass and are listed in Table 1 together with the suggested systematic compound name. Differences between theoretical and determined mass (Δm/z) as well as estimated physico-chemical properties for the suggested compounds are also shown in Table 1, while the suggested structures are shown in Fig. 2.

TP-214 had the same mass as OIT itself, as well as similar fragmentation pattern, but eluted at a different chromatographic retention time. This suggests photo isomerization as described by Rokach and Hamel^28^ for 2-substituted-isothiazol-3(2 *H*)-ones: after an initial break of the activated N-S-bond, the isothiazol-3(2 *H*)-one isomerized into the corresponding thiazol-2(3 *H*)-one (Fig. 2).

Additionally, the break of the N-S-bond led to the stepwise degradation of the ring, as the molecular ions of the remaining transformation products correspond to *N*-octylprop-2-enamide (TP-184a), *N*-octylacetamide (TP-172), *N*-octylformamide (TP-158) as well as octylamine (TP-130) as a final product.

A molecular ion of 216.1595 Da, which corresponds to *N*-octyl malonamic acid (TP-216), has been detected in very low intensity, which might be the initial intermediate of the isothiazolinone ring degradation. All previously mentioned transformation products were validated by analytical standards. Octylamine and *N*-octylacetamide were also hypothesised as a degradation product of DCOIT by Sakkas *et al*.^24^ however, they suggested different intermediate products, which were not detected in the present study. *N*-Octyl malonamic acid has been described as biotransformation product^27^, while the remaining four have not been described as transformation products of DCOIT so far. An overview of the proposed photodegradation pathways of OIT is presented in Fig. 2.

Based on molecular ion and fragmentation pattern, additional transformation products were suggested. Via some intermediate reaction steps, an S-O exchange might have led to the oxazolone derivate (TP-198). TP-184b might be the corresponding degradation product to TP-184a, *N*-ethenyl-*N*-octylformamide, deriving from the degradation of 2-octylthiazol-2(3 *H*)-one (TP-214). MS2-scans of those two compounds as well as suggested fragmentation pattern can be found in the supplementary material Figure S1. Other degradation products have been detected at 188.1646 Da (TP-188), 182.1533 Da (TP-182), and 144.139 Da (TP-144). In older studies acetic acid and formic acid were determined as degradation products of CMI and DCOIT^25^. However, as in the present study the lower limit of the MS-scan was set to 100 Da, they would not have been detected. *N*-octyl oxamic acid has been determined as degradation product (bio-/photodegradation, hydrolysis) in previous studies for DCOIT^26^ and was added in the following analysis.

The auxiliary kinetics experiments to get a first impression on the stability of OIT and its transformation products indicates that the photodegradation of OIT in tap water is following first-order kinetics (r^2^ = 0.9538) under the chosen conditions (tap water; 254 nm; 2.31*10^−10^ Einstein cm^−2^ s^−1^), with a half-life of 28 h and a photodegradation rate constant of 0.026 h^−1^ (Supplementary S2). Dark controls at 4 °C showed less than 10% degradation of OIT within one week. For the seven quantified degradation products 3-octylthiazol-2(3 *H*)-one, *N*-octylprop-2-enamide, *N*-octylacetamide, *N*-octylformamide as well as octylamine, *N*-octyl malonamic acid, and *N*-octyl oxamic acid maximum concentrations ranging from a few mg mL^−1^ (*N*-octylacetamide) to 2000 mg mL^−1^ (octylamine) were detected. Within 48 h of irrigation, the concentrations increased for all compounds besides 3-octylthiazol-2(3 *H*)-one. 3-Octylthiazol-2(3 *H*)-one peaked after about 10 h at 1000 mg mL^−1^, whereupon it was degraded as well (Supplementary S2). In the photodegradation experiments of OIT in solution, the concentrations of the seven validated compounds can explain more than 97% of the mass balance after 25 h irrigation; however, after 40 h they only accounted for 80% of the mass balance.

### Fate of OIT from artificial walls in the environment

#### Emissions of OIT and its degradation products

**Figure 3:**
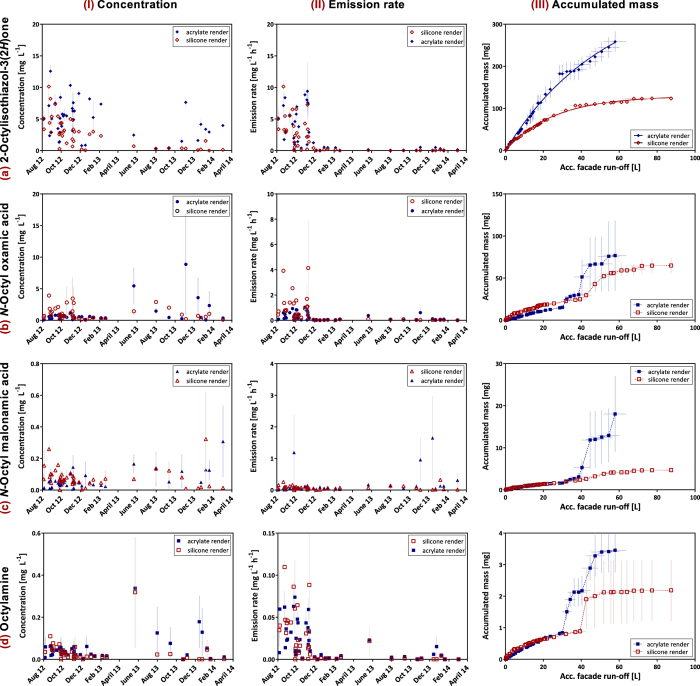
(**I**) Changes of (**a**) the OIT concentration and (**b**–**d**) its three most important transformation products in the run-off from artificial walls (1 × 1 m^2^) equipped with acrylate and silicone resin render over time, as well as (**II**) their emissions rates and (**III**) the accumulated emitted mass versus the accumulated run-off volume; error bars: standard error of mean from three panels.

Over the entire exposure time of 19 months, the concentrations of OIT in the wall run-off due to rain events ranged between below detection limit (<0.02 mg L^−1^) and 12 mg L^−1^ (silicone render) and 14 mg L^−1^ (acrylate render), respectively. The concentrations were varying considerably from event to event. However, a general decreasing trend is visible (Fig. 3.Ia). Six of the seven studied degradation products – octylamine, *N*-octyl oxamic acid, *N*-octyl malonamic acid, *N*-octylformamide, *N*-octylacetamide, and *N*-octylprop-2-enamide – were detectable in most of the samples in concentrations up to 8.8 mg L^−1^ (*N*-octyl oxamic acid). 3-Octylthiazol−2(3 *H*)-one was detected sporadically in concentrations up to 10 μg L^−1^. In general, there is no trend detectable for the concentrations of degradation products (Fig. 3.Ib-d).

Previous studies have shown that the leaching of biocides is correlating with the wind driven rain intensity^4^,^15^, i.e., the amount of rain hitting the facade (equals run-off volume) per duration of the respective rain event. In order to compare the release of OIT and its transformation products, emission rates (emitted mass of biocide per litre run-off per event duration) were calculated and are presented in Fig. 3.II. The emission rate of OIT was decreasing considerably from about 5 to 10 mg L^−1^ h^−1^ to below 1 mg L^−1^ h^−1^ within the first half year of exposure. The emission rates of the degradation products correlated with the emissions of OIT. However, while the OIT emission rate kept low, octylamine was emitted with slightly higher rates in the early summer 2013 (Fig. 3.IId), which might link to higher UV-degradation during this time. For *N*-octyl oxamic acid as well as *N*-octylformamide (not shown) higher emission rates were detected from silicone render within the first half year of exposure compared to acrylate render, while for the other compounds comparable emission rates were measured.

When considering the accumulated emitted mass in dependency of the accumulated run-off (Fig. 3.IIIa), it appears that OIT emissions are more and more decreasing over time, following an exponential function (Eq. 1) with *M*_*Acc*_ being the accumulated emitted mass, *M*_*Max*_ the (asymptotic) maximum accumulated emitted mass, *K* the emission rate constant and *V*_*run-off*_ the accumulated run-off volume.


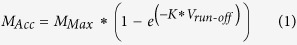


Especially in case of silicone render equipped panels, nearly no OIT is released after about 40 L m^−2^ run-off. In contrary, the emissions of the transformation products were not following an easily recognizable pattern.

#### Mass balance of OIT on renders

The mass balance for OIT on the walls cannot be closed with the presented data, as even including seven transformation products, the mass balance only adds up to 30–40% on acrylate and silicone render, respectively. To make matters worse, the material also contained DCOIT, which can photodegrade following a similar degradation pattern (e.g. octylamine and *N*-octyl malonamic acid described by Sakkas *et al*.^24^).

**Figure 4:**
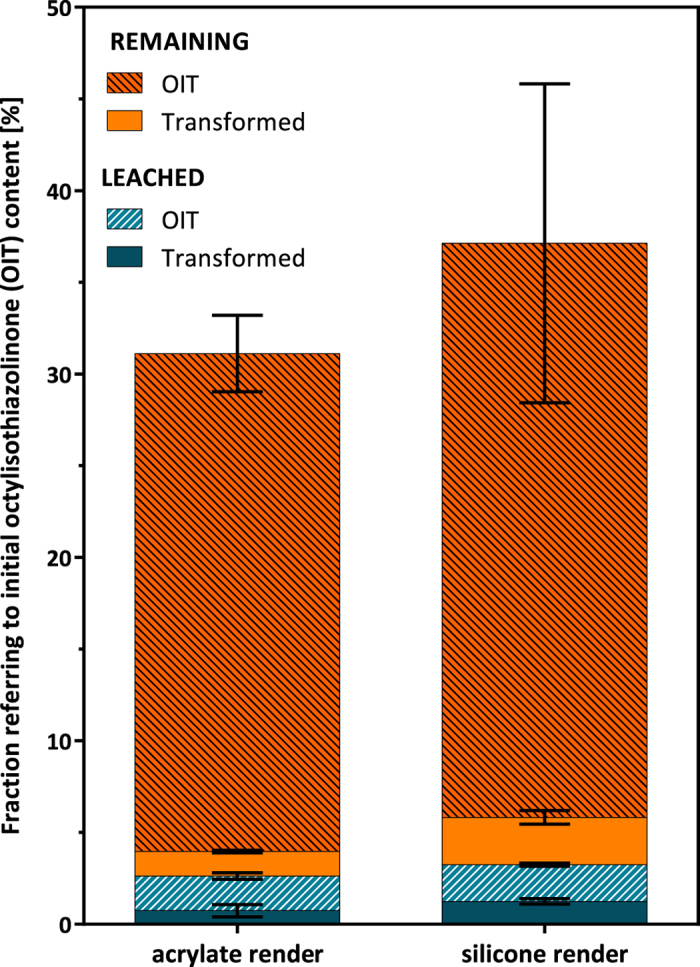
Mass balance of OIT: leached OIT [OIT detected in run-off water (average from 3 panels)], leached transformed [sum of transformation products detected in run-off (average from 3 panels)], remaining OIT [OIT in render after 19 month exposure (average of 10 extracts from one panel)], remaining transformed [sum of transformation products in render after 19 month exposure (average of 10 extracts from one panel)]; error bars: standard error of mean.

As shown in Fig. 4, the majority of OIT was still remaining in the coating after 19 months of exposure to natural weather (27% acrylate render; 31% silicone render) followed by the OIT fraction leached (2%). The total fraction of transformation products detected in run-off or remaining in the coating was 2% (acrylate) and 4% (silicone), respectively. It is noticeable, that the fraction of transformation products remaining in the material was twice as much as the fraction of transformation products leached. However, the uneven distribution was not detectable for the most important degradation product *N*-octyl oxamic acid, with 1.1% (silicone) and 0.6% (acrylate) leached and 1.4% (silicone) and 0.6% (acrylate) remaining in the material.

## Discussion

With a photodegradation half-life of 28 h in solution while irradiated with 2.31*10^−10^ Einstein cm^−2^ s^−1^, the degradation rate of OIT is comparable to the chlorinated analogue DCOIT^24^. As known for other isothiazol-3(2 *H*)-ones, the photodegradation of OIT is also initiated by a cleavage of the ring structure. However, although being the most important transformation product in previous studies^24^,^25^, *N*-octyl malonamic acid is not or only tentatively detected in the present study at very low concentrations. This might result from the monochromatic light source (254 nm) or another possibility is that the degradation of *N*-octyl malonamic acid is much faster than its formation.

The long-term emissions from biocide-equipped polymer resin render showed that OIT is leaching predominantly within the first half year after application. However, although the emissions decrease, a huge fraction of OIT is still detectable in the material. This raises the question on the limiting processes on the transport of the active substances within the material. For the first months a linear correlation between leached amount and run-off is visible, which suggests that only those biocide molecules that are easily in contact with water (outer and inner surfaces / surface layer) are leaching with time. The refill of this surface layer by diffusion through solid material seems to be too slow to equilibrate rapidly. This division of the process into two phases is supporting the suggestion proposed by Wittmer *et al*.^30^ a fast and a slow diffusion mechanism. Also the render composition, namely the organic resin binder, seems to have an influence: for most of the events the OIT concentrations in the leachates from the acrylate renders was considerably higher than those from the silicone renders, which was not only an artefact of the higher run-off volume from silicone render. As the exponential functions of the accumulated mass of OIT emitted to the leachate are reaching a plateau, maximum endpoints of 2.1% and 2.8% of the initial OIT amount can be expected for silicone and acrylate render, respectively.

By including seven degradation products (octylamine, *N*-octyl oxamic acid, *N*-octyl malonamic acid, *N*-octylformamide, *N*-octylacetamide, *N*-octylprop-2-enamide and 3-octylthiazol-2(3 *H*)-one) in the analysis of wall run-off as well as the remaining fraction in the render, the mass balance of OIT in facade coatings is still showing gaps of 60–70%. This is also in accordance with the results from the degradation kinetic study in solution: while at earlier time points the mass balance can be closed, the mass balance after 48 h irradiation showed a gap of 23%. However, this gap in the mass balance is in contrast to previous findings for terbutryn on façade render, where the mass balance could be closed by the inclusion of transformation products^21^. These gaps in the OIT mass balance might be due to either vaporisation or reactions to undetected transformation products. While vaporisation of OIT is expected to be low^31^, the most important degradation product octylamine is considerably more volatile in comparison (vapour pressure ρ_vap_: 131 Pa, i.e., about 20000 times higher than OIT). Hence, emissions to the air cannot be excluded. Additionally, complete mineralization via acetic acid and formic acid, as described in earlier studies for MI or DCOIT^24^,^25^, was not included in our measurements. Products resulting from reactions with OH-radicals were most probably covered based on the laboratory experiments. However, reactions with other reactive atmospheric species as N_X_O_Y_-radicals or ozone might have formed other undetermined products. One could also imagine that activated OIT species have formed molecular bonds with the polymer matrix. 2-Octylcarbamoyl-1-ethene sulfonic acid has been determined as degradation product (bio-/ photodegradation, hydrolysis) in previous studies on DCOIT^26^. As no standard was available at this time, low resolution suspect screening for this compound was performed which revealed, that it might be present in the run-off samples and render extracts from the panel study (see Supplementary S1), while it was not present in the UV-degradation study.

In conclusion, the mass balance for OIT in polymer resin renders cannot be closed at this time. Nevertheless, the study showed that for the assessment of the fate of biocides in coating material, transformation products need to be taken into account. This goes for the emissions via the runoff but also the remaining in the material. Furthermore, in case of volatile degradation products, also the emissions to air should not be neglected, especially in indoor environments where effects on health are of high concern.

## Materials and methods

### Identification of photodegradation products with HR-MS

100 mL of an aqueous OIT solution (8 μg mL^−1^, tap water with 1% methanol) were irradiated with monochromatic UV-light (three 254 nm-lamps of 5 W) for 48 h. The irradiation resulted in a radiation intensity of 2.3*10^−10^ Einstein cm^−2^ s^−1^ (1.1–1.3 W m^-2^) at the water surface. The setup was located in a temperature controlled chamber and the samples were cooled during irradiation as described previously by Minelgaite, *et al*.^32^. Samples were taken at different time points (0 h, 5 h, 10 h, 20 h, 25 h, 30 h and 48 h). Afterwards the samples were stored at 4 °C until analysis. A dark control experiment was performed at 4 °C.

The identification of degradation products was performed on an HPLC (1200 Series, Agilent Technologies, Waldbronn, Germany) coupled to a QToF-MS (TripleToF 5600, AB Sciex, Framingham, MA, USA) according to Schlüsener, *et al*.^33^. The separation was performed on an Agilent Zorbax Eclipse Plus C18 Narrow Bore RR (2.1 × 150 mm; 3.5 μm) using a water-acetonitrile (both with 0.1% formic acid) multistep gradient. The QToF-MS was operated in full scan mode (100–1200 Da), with automatic product ion scans (30–1200 Da) on all peaks exceeding 100 cps. Both positive and negative mode acquisitions were performed. Afterwards standards were purchased for the suggested compounds (suppliers see supplementary material S3) and validated and quantified by triple quadrupole mass spectrometry (see below) using retention time and product ion scans.

### Construction of panels

Six panels were constructed according to usual construction practice for external thermal insulation composite systems (ETICS). A 1 × 1 m polystyrene plate was attached on a wooden plate in a metal frame 1 m above the ground. A base coat with fiberglass reinforcement mesh was applied which was then coated with acrylate (N = 3) and silicone (N = 3) final top render, respectively. For the final top render, two for the building market typical commercial products were used after modifications (acrylate resin render: KHK, Quick-Mix, Osnabrück, Germany; silicone resin render: HECK SHP KC1, BASF Wall Systems, Marktredwitz, Germany). Prior to the application of the final topcoat (render), OIT was added as an industrial suspension and stirred well. Final concentrations in the render were 4.6 g kg^−1^ (acrylate render) and 3.3 g kg^−1^ (silicone render). Due to different application thicknesses the total amount applied per panels was 13.6 g m^−2^ (acrylate render) and 6.2 g m^−2^ (silicone render). Due to the experimental setup the materials contained similar amounts of other isothiazolinones (MI, BIT, DCOIT) as well.

### Sampling and analysis

The panels were exposed to natural weather between August 2012 and March 2014, facing south-west (predominant wind direction in Denmark). A metal rain gutter in the bottom of the panel was connected to a 2 L-glass bottle, which was exchanged after every rain event. The total run-off of about 80 L, equal to 70 rain events, was collected and analysed. While the first 30 events were analysed separately, events 31 to 70 were analysed as composite samples of about 5 L run-off each. The pH of the run-off water was occasionally tested to be around 6.

For the analysis of OIT the first samples were diluted with methanol (events 1–14 1:100, events 15–54 1:10), while the samples from events 55–70 were analysed directly. For transformation products all samples were analysed directly. To 1 mL of (diluted) sample 50 μL surrogate standard (OIT-D_17_, 1 μg mL^−1^ in methanol (LiChrosolv gradient grade, Merck, Darmstadt, Germany)) was added and analysed by high performance liquid chromatography with tandem mass spectrometry (HPLC-MS/MS) using electrospray ionization in positive mode (ESI(+)) on an Ultimate 3000 dual gradient low pressure mixing HPLC-system (Dionex, Sunnyvale, CA, USA) coupled to an API 4000 triple-quadrupole-MS (AB Sciex, Framingham, MA, USA). OIT was analysed according to Bollmann *et al*.^4^ while the transformation products were analysed using an acidified water - acetonitrile multistep gradient. The used gradient as well as precursor and products ions can be found in the supplementary material (Supplementary S3 and S4).

### ASE extraction of biocides from render material

After 19 months of exposure, one acrylate and one silicone render panel were sampled for the remaining fraction of OIT. 10 subsamples (top coat together with base coat, ~ 60 cm^2^ each) were taken, representing an even distribution across the panel, and crushed in a mortar. 1 g of each subsample, mixed with 1.5 g Hydromatrix, was extracted by accelerated solvent extraction (ASE 200, Dionex, Sunnyvale, CA, USA) in duplicate. Free space in the 11 mL cells was filled up with Ottawa-Sand. The cells were extracted at 110 °C and 1600 psi (110 bar), using methanol (LiChrosolv gradient grade, Merck, Darmstadt, Germany) as a solvent. In total 3 extraction cycles were performed with the following settings: static time 5 min, preheating time 1 min, flush 60%, purge 60%. Afterwards, 500 μL extract was diluted with 1 mL methanol, spiked with 50 μL surrogate standard (OIT-D_17_, 1 μg mL^−1^ in methanol) and analysed by HPLC-MS/MS as earlier described. Extraction recoveries ranged between 35% and 62% with standard deviations below 5% and were used for correction of the results (Supplementary S5).

### Weather during the sampling period

A weather station 900 m linear distance away provided data for rain quantity and duration, humidity, temperature, radiation, pressure, wind speed and direction data at 2 m height, corresponding to the elevation of the panels above ground^34^. In total 1044 mm rain was recorded within the sampling period, of which 277 mm fell within the first 30 events. Average wind direction and speed during the first 30 rain events was SSW (202.5°) and 6 m s^−1^. The radiation maxima during day time varied between 150 W m^−2^ (December/January) and 950 W m^−2^ (June/July). Two heavy storm events occurred: St Jude storm/ Cyclone Christian on the 28 October, 2013 and Cyclone Xaver on the 6 December, 2013 with wind speeds >100 km h^−1^. During the first storm, three panels toppled over (one acrylate, two silicone panels). However the sampling bottles stayed, so that samples could be analysed, while all bottles were destroyed during the second storm. A complete weather data overview is available in the supplementary material S6.

## Additional Information

**How to cite this article**: Bollmann, U. E. *et al*. Photodegradation of octylisothiazolinone and semi-field emissions from facade coatings. *Sci. Rep.* **7**, 41501; doi: 10.1038/srep41501 (2017).

**Publisher's note:** Springer Nature remains neutral with regard to jurisdictional claims in published maps and institutional affiliations.

## Supplementary Material

Supplementary Information

## Figures and Tables

**Figure 1:**
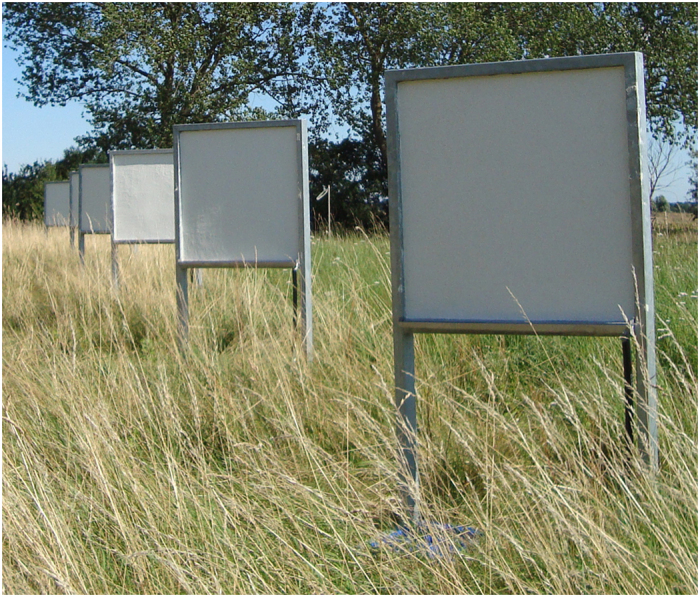
Experimental setup: artificial walls (1 × 1 m) for field emissions.

**Table 1:** OIT photodegradation products: detected molecular ions in HR-MS (ESI+) with deviation of measured versus theoretical mass (Δm/z), product ion spectra, compound name, chemical formula, validation with analytical standard (yes/no), and estimated physico-chemical properties (WS: water solubility; Log K_OW_: octanol-water partition coefficient, ρ_vap_: vapour pressure, Log K_OA_: octanol-air partition coefficient).

Abbr.	RT [min]	Detected m/z Δm/z [Da]	Product ion spectrum m/z [Da] CE: 40 V	Compound Chemical formula	Validation analytical standard	Properties (calc. with EPI suite^35^)
OIT	10.12	214.1259–0.0007	102.0023,57.0749, 43.0615, 83.9922	2-Octylisothiazol-3(2 *H*)-one C_11_H_19_NOS	yes	WS: 302 mg L^−1^ Log K_OW_: 2.61 ρ_vap_: 0.0266 Pa Log K_OA_: 8.5
TP-214	14.07	214.1252–0.004	102.0020, 77.0413, 141.0004, 57.0737, 43.0601	3-Octylthiazol-2(3 *H*)-one C_11_H_19_NOS	yes	WS: 27 mg L^−1^ Log K_OW_: 3.7 ρ_vap_: 0.0031 Pa Log K_OA_: 6.3
TP-158	11.06	158.1527–0.0018	41.0444, 43.0599, 46.0332, 57.0732, 39.0293, 77.0399, 71.0878, 60.0478	*N*-Octylformamide C_9_H_19_NO	yes	WS: 776 mg L^−1^ Log K_OW_: 2.29 ρ_vap_: 0.0798 Pa Log K_OA_: 7.3
TP-184a	12.03	184.17010	55.0210, 72.0459, 43.0593,	*N*-Octylprop-2-enamide C_11_H_21_NO	yes	WS: 120 mg L^−1^ Log K_OW_: 3.1 ρ_vap_: 0.013 Pa Log K_OA_: 8.5
TP-172	11.14	172.1696–0.0005	60.0470, 57.0722, 41.0453, 89.9402, 112.9550	*N*-Octylacetamide C_10_H_21_NO	yes	WS: 276 mg L^−1^ Log K_OW_: 2.74 ρ_vap_: 0.031 Pa Log K_OA_: 7.9
TP-130	6.95	130.1594–0.0004	41.0461, 43.0612, 39.0304, 57.0742, 71.0880	Octylamine C_8_H_19_N	yes	WS: 3147 mg L^−1^ Log K_OW_: 2.8 ρ_vap_: 131 Pa Log K_OA_: 4.4
TP-216	10.26	216.1595–0.0005	n.a.	*N*-Octyl malonamic acid C_11_H_21_NO_3_	yes	WS: 720 mg L^−1^ Log K_OW_: 2.0 ρ_vap_: 5.75 10^–5^ Pa Log K_OA_: 12.3
TP-198	12.85	198.1486–0.0008	86.0251, 57.0732, 43.0592, 41.0442	2-Octyl-1,2-oxazol-3(2 H)-one?3-Octyl-1,3-oxazol-2(3 H)-one?	no	
TP-184b	11.28	184.17–0.0001	72.0471, 41.0459, 43.0598, 166.1592	*N*-ethenyl-*N*-octylformamide? C_11_H_21_NO	no	
TP-188	7.36	188.1646	44.0546, 43.0596, 57.0737, 71.0876, 142.1587, 119.9421		no	
TP-182	12.48	182.1533	53.0059, 70.0309, 57.0737, 43.0595		no	
TP-144	9.16	144.139	41.0434, 43.0608, 57.0732, 60.9893, 74.0609, 88.0769		no	
