# Supplementary material for: Photodegradation of octylisothiazolinone and semi-field emissions from facade coatings
Source: Sci Rep. 2017 Jan 27;7:41501. doi: 10.1038/srep41501 (PMC5269726; doi:10.1038/srep41501)

Photodegradation of octylisothiazolinone and semi-field emissions from facade coatings

→ Supplementary material

Ulla E. Bollmann[1]*, Greta Minelgaite[2], Michael Schlüsener[3], Thomas A. Ternes[3], Jes Vollertsen[2], Kai Bester[1]

[1] Aarhus University, Department of Environmental Science, Frederiksborgvej 399, 4000 Roskilde, Denmark

[2] Aalborg University, Department of Civil Engineering, Sofiendalsvej 11, 9200 Aalborg SV, Denmark

[3] German Federal Institute for Hydrology, Am Mainzer Tor 1, 56068 Koblenz, Germany

Table of content

Supplementary S1. MS^2 spectra (ESI+, CE = 40 V), chemical structures and suggested fragmentation pattern of (1a) TP-184b, (1b) TP-198, and (2) TP-264.

Supplementary S2. Photodegradation of OIT dissolved in tap water under UV-light and formation of degradation products over time.

Supplementary S3. Mass spectrometric data and suppliers for OIT and its degradation products.

Supplementary S4. Chromatographic gradient for the analysis of OIT transformation products.

Supplementary S5. Extraction recoveries from render using accelerated solvent extraction.

Supplementary S6. Weather data for field experiments (data points: 10 min average).

Supplementary S1-1. MS^2 spectra (ESI+, CE = 40 V, TripleToF 5600), chemical structures and suggested fragmentation pattern of (a) TP-184b (suggestion: *N*-ethenyl-*N*-octylformamide) and (b) TP-198 (suggestion: 3-octyl-1,3-oxazol-2(3*H*)-one) from the HR-MS analysis of the laboratory degradation study.

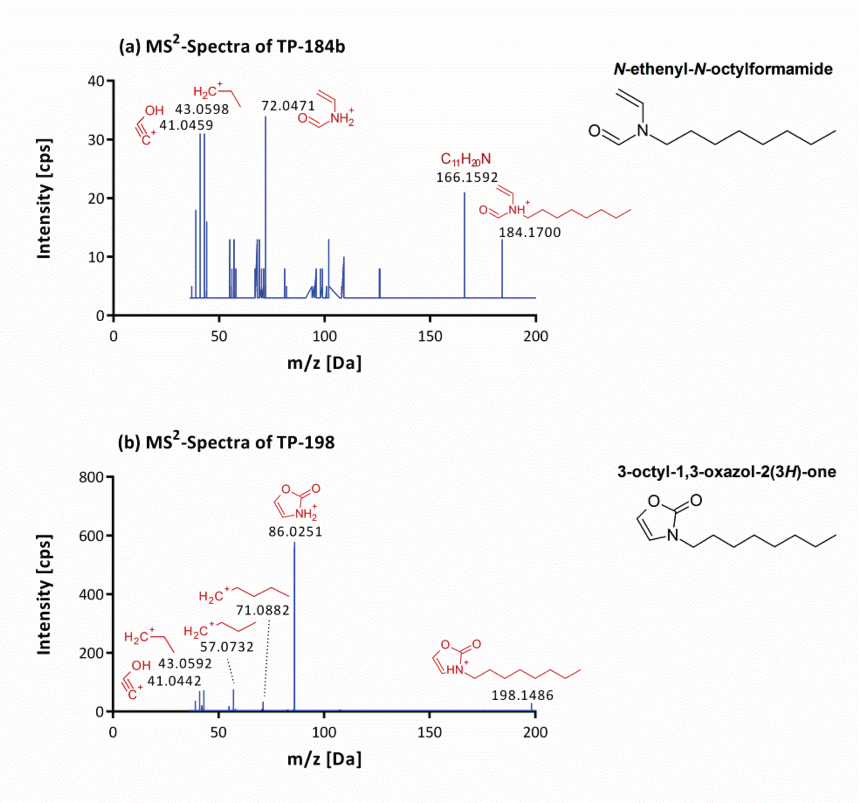

Supplementary S1-2. MS^2 spectra (ESI+, CE = 40 V, API4000), chemical structures and suggested fragmentation pattern of TP-264 (suggestion: 2-octylcarbamoyl-1-ethene sulfonic acid) detected in a suspect screening in runoff samples and extracts of the render of the studied panels.

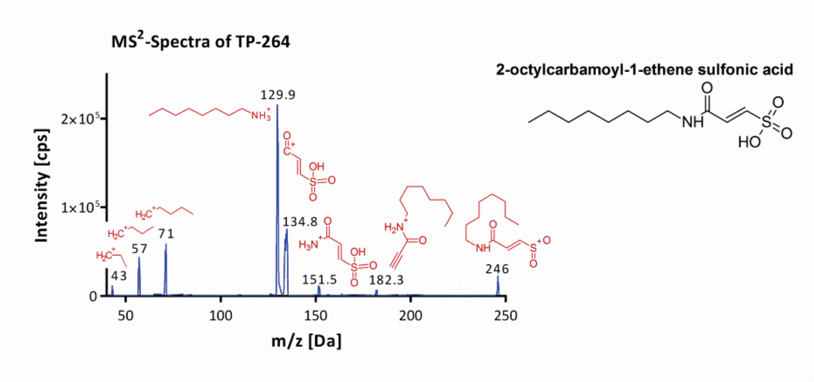

Supplementary S2. Photodegradation of OIT (regression: first-order kinetic, r^2= 0.9538) dissolved in tap water under UV-light (254 nm; $2.31*10^{-10}$ Einstein cm^{-2} s^{-1}) and formation of degradation products over time (error bars: standard error of the mean of two replicates).

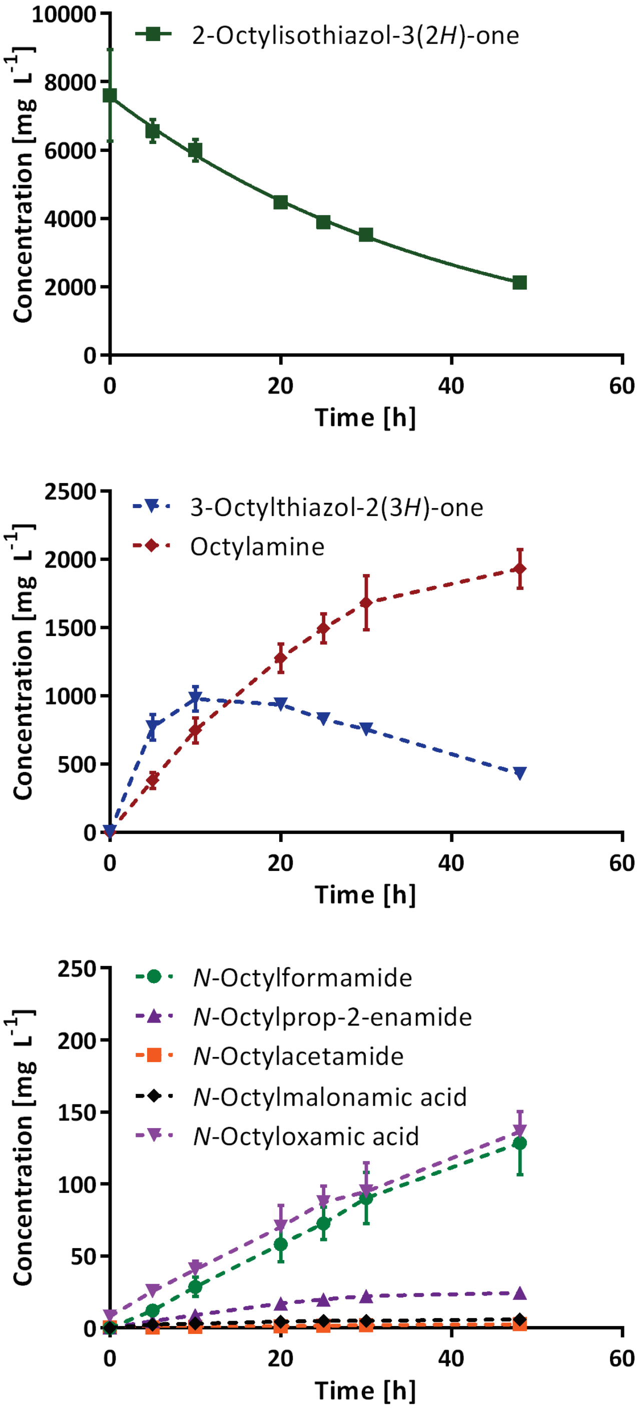

Supplementary S3. Mass spectrometric data and suppliers for OIT and its degradation products.

ID	Name	Supplier	Precursor	Product	DP	EP	CE	CXP
OIT	2-Octylisothiazol-3(2*H*)-one	Sigma-Aldrich	214	102	55	10	22	19
				57	84	10	30	10
OIT-D_{17}	2-Octylisothiazol-3(2*H*)-one-D_{17}	TRC	231	103	47	10	22	5
				66	54	10	34	12
TP-214	3-Octylthiazol-2(3*H*)-one	Uorsy	214	57	84	10	30	10
				57	60	10	35	9
TP-158	*N*-Octylformamide	TCI-Chemicals	158	57	68	10	25	10
				71	64	10	19	12
TP-184a	*N*-Octylprop-2-enamide	Uorsy	184	55	61	10	42	9
				43	45	10	41	7
TP-172	*N*-Octylacetamide	Uorsy	172	60	61	10	26	12
				43	69	10	44	7
TP-130	Octylamine	Sigma-Aldrich	130	57	60	10	25	12
				71	48	10	22	12
TP-216	*N*-Octyl malonamic acid	Chemspace	216	71	48	10	27	13
				156	50	10	17	9
TP-202	*N*-Octyl oxamic acid	Chemspace	202	71	48	10	17	13
				156	45	10	13	9

Supplementary S4. Chromatographic gradient for the analysis of OIT transformation products.

Water/0.2% formic acid (A) and acetonitrile/0.2% formic acid (B) gradient for octylamine, *N*-octylformamide, *N*-octylprop-2-enamide, *N*-octylacetamide & 3-octylthiazol-2(3*H*)-one: flow 300 µL min^{-1}; gradient: 0-1 min 2% B, 1-2 min 2→40% B, 2-10 min 40→75% B, 10-10.5 min 75→100% B, 10.5-12 min 100% B, 12-12.5 min 100→2% B, 12.5-15 min 2% B. Column: Synergy Polar-RP column, L = 150 mm, ID = 2 mm, particles = 4 µm, 5°C (Phenomenex, Torrance, CA, USA).

Water/0.2% formic acid (A) and methanol/0.2% formic acid (B) gradient for *N*-octyl oxamic acid & *N*-octyl malonamic acid: flow 250 µL min^{-1}; gradient: 0-1 min 0% B, 1-4.5 min 0→100% B, 4.5-6.5 min 100% B, 6.5-6.8 min 100→0% B, 6.8-12.5 min 0% B. Column: Kinetiex PFP column, L = 50 mm, ID = 2.1 mm, particles = 2.6 µm, 25°C (Phenomenex, Torrance, CA, USA).

Supplementary S5. Extraction recoveries from render using accelerated solvent extraction (N=6).

Name	Recovery (Std Dev.) [%]
2-Octylisothiazol-3(2*H*)-one	43 (5)
3-Octylthiazol-2(3*H*)-one	55 (0.8)
N-Octylformamide	54 (0.9)
N-Octylprop-2-enamide	62 (1)
N-Octylacetamide	59 (0.9)
Octylamine	46 (1)
N-Octyl malonamic acid	36 (4)
N-Octyl oxamic acid	35 (4)

Supplementary S6. Weather data for field experiments (data points: 10 min average).

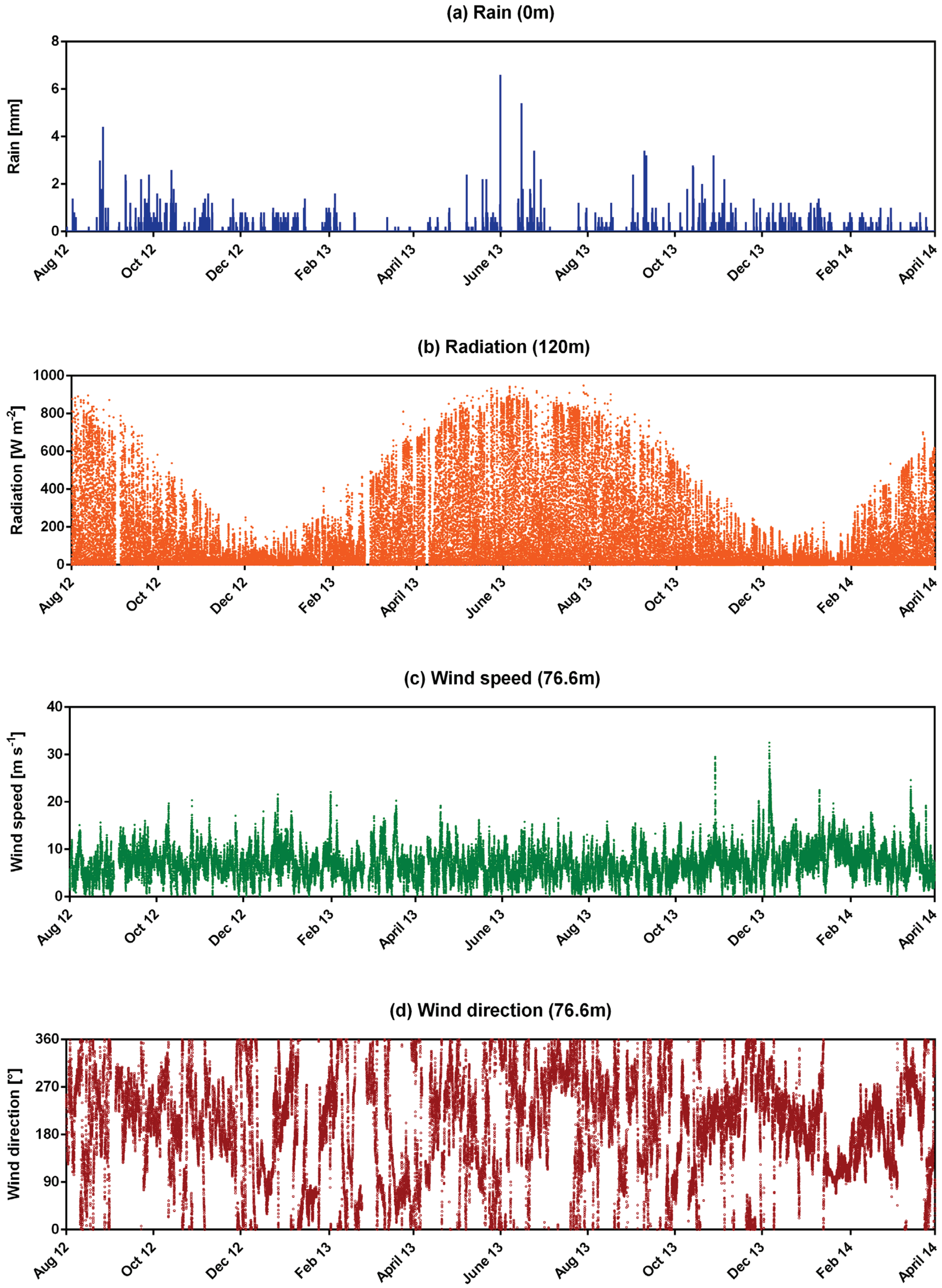

Supplement: Supplementary Information [file srep41501-s1.pdf]
